# Beyond Pressure Gradients: The Effects of Intervention on Heart Power in Aortic Coarctation

**DOI:** 10.1371/journal.pone.0168487

**Published:** 2017-01-12

**Authors:** Joao Filipe Fernandes, Leonid Goubergrits, Jan Brüning, Florian Hellmeier, Sarah Nordmeyer, Tiago Ferreira da Silva, Stephan Schubert, Felix Berger, Titus Kuehne, Marcus Kelm, on behalf of the CARDIOPROOF Consortium

**Affiliations:** 1 Department of Congenital Heart Disease, German Heart Centre Berlin, Augustenburger Platz 1, Berlin, Germany; 2 Biofluid Mechanics Laboratory, Charité–Universitätsmedizin Berlin, Berlin, Germany; 3 Pediatric Cardiology, Charite–Universitätsmedizin Berlin, Berlin, Germany; 4 DZHK (German Centre for Cardiovascular Research), partner site Berlin, Berlin, Germany; UMCU, NETHERLANDS

## Abstract

**Background:**

In aortic coarctation, current guidelines recommend reducing pressure gradients that exceed given thresholds. From a physiological standpoint this should ideally improve the energy expenditure of the heart and thus prevent long term organ damage.

**Objectives:**

The aim was to assess the effects of interventional treatment on external and internal heart power (EHP, IHP) in patients with aortic coarctation and to explore the correlation of these parameters to pressure gradients obtained from heart catheterization.

**Methods:**

In a collective of 52 patients with aortic coarctation 25 patients received stenting and/or balloon angioplasty, and 20 patients underwent MRI before and after an interventional treatment procedure. EHP and IHP were computed based on catheterization and MRI measurements. Along with the power efficiency these were combined in a cardiac energy profile.

**Results:**

By intervention, the catheter gradient was significantly reduced from 21.8±9.4 to 6.2±6.1mmHg (p<0.001). IHP was significantly reduced after intervention, from 8.03±5.2 to 4.37±2.13W (p < 0.001). EHP was 1.1±0.3 W before and 1.0±0.3W after intervention, p = 0.044. In patients initially presenting with IHP above 5W intervention resulted in a significant reduction in IHP from 10.99±4.74 W to 4.94±2.45W (p<0.001), and a subsequent increase in power efficiency from 14 to 26% (p = 0.005). No significant changes in IHP, EHP or power efficiency were observed in patients initially presenting with IHP < 5W.

**Conclusion:**

It was demonstrated that interventional treatment of coarctation resulted in a decrease in IHP. Pressure gradients, as the most widespread clinical parameters in coarctation, did not show any correlation to changes in EHP or IHP. This raises the question of whether they should be the main focus in coarctation interventions. Only patients with high IHP of above 5W showed improvement in IHP and power efficiency after the treatment procedure.

**Trial Registration:**

clinicaltrials.gov NCT02591940

## Introduction

Although differences in international guideline treatment criteria exist, decision making in coarctation of the aorta (CoA) is regularly based on pressure gradients (pressure drops) [1, 2]. Additionally, a more general goal in cardiologic interventions and surgical procedures is to optimize the energetical state of the heart muscle allowing the most efficient oxygen consumption of the myocardial tissue. This goal can usually be reached by optimization of the hemodynamic situation [3–5]. However, in patients with CoA, even years after successful treatment of the narrowing, increased morbidity and mortality have remained major issues. In a broad age range various problems exist, including (exercise) hypertension and heart failure, and are not yet sufficiently understood [6–10].

Predictions concerning individual disease progression are not yet available and only little is known about energetical aspects of the disease. Moreover, current guidelines in CoA cannot address satisfactory methods of preventing long-term morbidity and mortality in treated patients, raising the question of whether the pressure gradient is a sufficient guide for energetical optimization in patients with CoA. Additionally, real peak-to-peak pressures are not available when the decision is made whether or not an invasive heart catheter procedure, with all its potential risks, should be performed. Non-invasive assessment methods are used instead during clinical routine including echocardiography and peripheral blood pressure measurements.

While analyses of the external heart power (EHP) and heart work (pressure-volume loop) have been performed before [11, 12], only little is known about internal heart power (IHP)–the energy the heart muscle needs to pump blood flow against a given pressure (afterload). Wall stress is one component of IHP and was recently proposed as a diagnostic method in heart failure [13].

We aimed to assess the effects of interventional treatment procedures on heart power in patients with CoA and to investigate the correlation of these parameters to commonly used peak-to-peak pressure gradients obtained from heart catheterization.

## Materials and Methods

### Study population and design

**Fig 1:**
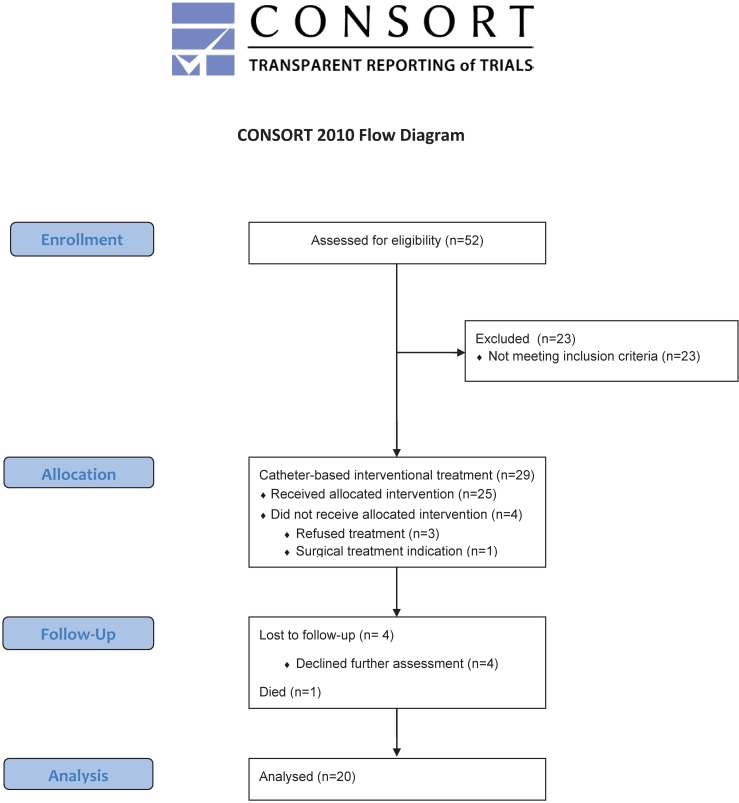
Study flow diagram. Flow of participants through the study.

In a prospective clinical study design 52 subsequent patients were identified who met the inclusion criteria of the aortic coarctation arm of the CARDIOPROOF trial (NCT02591940). All patients underwent specific MRI examinations at the German Heart Center Berlin (Deutsches Herzzentrum Berlin, DHZB) between December 2013 and December 2015. During the initial assessment peripheral blood pressure measurements were acquired during the MRI. The study flow diagram is shown in Fig 1.

In 29 of these patients an indication for interventional treatment was found. The indications included pressure gradients >20 mmHg, and/or arterial hypertension (according to percentiles for pediatric patients from Neuhauser et al. [14] and classification by the American Heart Association (AHA) guidelines for adult patients [15]) and/or severe narrowing. Overall 25 patients underwent a catheter-based interventional treatment procedure according to international standards.

The primary endpoints of the study were the assessment of internal and external heart power (IHP, EHP) and the power efficiency of the left ventricle after treatment. The secondary endpoints were (i) peak-to-peak pressure gradients, and (ii) the presence of arterial hypertension after treatment (S1 Table).

A total number of 20 patients (16 males/4 females) with a mean age of 20±14years (range 6 to 57years) underwent a complete follow-up assessment in median 4 days after treatment, including an MRI scan and clinical data collection in conjunction with blood pressure measurements until December 2015. These patients were included in the comparative heart power analysis. Measurements were performed after pseudo-anonymization in order to mask procedures to the observers. The baseline data is shown in Table 1.

**Table 1:** Summary of statistics at baseline for participants undergoing the comparative heart power analysis.

	Patient characteristics at baseline (*n = 20*)
**Subjects**	
Male gender, n (%)	16 (80%)
Age (years)	20±14 range [6–57]
BMI (kg/m^2^)	21.36±5.42
BSA (m^2^)	1.59±0.42
**Previous treatment**	
Native to treatment	5 (25%)
Surgical repair of CoA	7 (35%)
• Of those with previous catheter intervention	5 (71%)
Catheter intervention	12 (60%)
• Of those exclusively angioplasty	4 (33%)
• Of those exclusively stenting	1 (8%)
• Of those angioplasty and stenting	7 (59%)
**Associated conditions**	
Bicuspid aortic valve, n (%)	8 (40%)
Arterial hypertension	13 (65%)

The study was carried out according to the principles of the Declaration of Helsinki and approved by the local ethics committee (Ethics committee—Charité—Universitätsmedizin Berlin, S1 and S2 Protocols). Written informed consent was obtained from the participants and/or their guardians.

### MRI data acquisition

All MRI examinations, were conducted in a whole body 1.5 Tesla MR system (Achieva R 3.2.2.0, Philips Medical Systems, Best, The Netherlands) using a five-element cardiac phased-array coil. MRI scans were performed between September 2013 and October 2015. Blood pressure was further measured just after the MRI scan using the Riva-Rocci (RR) method: systolic/diastolic (mean). All examinations were conducted successfully and lasted between 45 and 60 minutes. None of the subjects reported any clinical symptoms attributable to MRI.

Three-dimensional anatomy of the ventricles was determined by balanced turbo field echo (bTFE) cine two-dimensional sequences in short axis. For each image sequence one heartbeat was divided into 25 time-steps forming the cine image. The typically used sequence parameters were: echo time 1.96ms, repetition time 3.97ms; flip angle 60°, field of view 420mm x 330mm; parallel imaging with an acceleration factor of 2 (SENSE); voxel size 1.5mm x 1.5mm and slice thickness 7 mm.

Using previously established protocols [16] flow rates in the ascending aorta were measured using 4D-VEC MRI of the thorax. An anisotropic 4D segmented k-space phase contrast gradient echo sequence with retrospective electrocardiographic gating but without navigator gating of respiratory motion was used to minimize acquisition time. The sequence parameters were: matrix size 100 x 128, 30 slices, acquired voxel 2.5 x 2.5 x 2.5mm, reconstructed voxel 1.7 x 1.7 x 2.5mm, TR 3.5msec, TE 2.2msec, FA 5°, 25 reconstructed cardiac phases, velocity encoding 4.0 m/s, number of signal averages 1. Scan time varied between 9 and 14 minutes, depending on the size of the patient’s chest. The high velocity encoding in all three directions was used in order to avoid phase wraps in the presence of stenosis forming complex 3D flow.

ZIBAmira (Zuse Institute Berlin, Germany) was used for analysis of the anatomy: the acquired images were resampled to refine spatial resolution. The end-systolic blood pool and myocardium were segmented and myocardial inner and outer surfaces were generated. From the 3D generated left ventricle masks myocardium wall thickness, blood pool and myocardium volumes were calculated. Post-processing for analysis of flow rates across the aortic valve was carried out with GTFlow 1.6.8 software (Gyrotools, Zurich, Switzerland).

### Catheterization and intervention

During catheterization pressure curves were obtained over the cardiac cycle in the ascending aorta and the left ventricle (LV) before treatment, and repeated in the ascending aorta after an interventional treatment procedure was performed. Contraction time was measured from LV pressure curves using a MATLab® (R2012a, The MathWorks Inc., Natick, MA, USA) script in order to detect the time period between the first pressure increase and the peak systolic pressure.

Pressures were recorded simultaneously between three predefined locations (left ventricle, the ascending and the descending aorta) and the femoral artery during cardiac catheterization. Patients were sedated by intravenous administration of a bolus of midazolam (0.1–0.2 mg/kg, max. 5 mg), followed by a bolus of propofol (1–2 mg/kg, as needed) and continuous infusion of propofol (approximately 4 mg/kg/h, as needed). Pressure measurements were taken with senior cardiologists present. Pigtail catheters (Cordis, Warren, NJ, USA) of 5-6F were connected to pressure transducers (Becton-Dickinson, Franklin Lakes, NJ, USA). Routinely, patients received balloon angioplasty with or without additional placement of a stent in order to treat a given stenosis by removing the narrowing of the vessel and thus the pressure gradient. All procedures were performed by an experienced interventionalist. In order to reduce cardiac catheterization duration, only the ascending aorta pressures were measured post-treatment. The Schwarzer hemodynamic analyzing system (Schwarzer, Heilsbronn, Germany) was used to amplify, acquire, and analyze the pressure signals. Duration of the cardiac catheterization is on average 1 hour.

To export all measured data (pressures and ECG) from a binary format an in-house MATLab script was developed allowing automatic analysis of the heart rate and detection of the local peaks and valleys. These values were used to measure contraction time, peak systolic pressure in the left ventricle and in the ascending aorta, and end-diastolic left ventricle pressure.

### Cardiac energy profile

**Fig 2:**
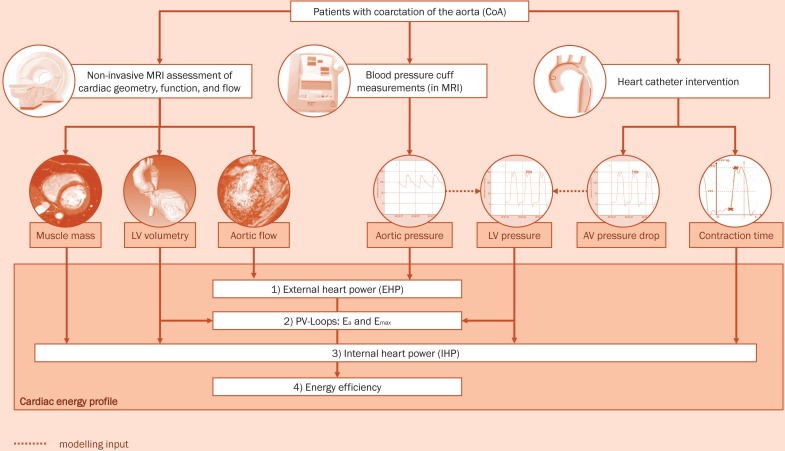
The assessment of a cardiac energy profile in patients with aortic coarctation. In all evaluated study participants this profile was acquired before and after the interventional treatment procedure. AV Aortic valve, CoA coarctation of the aorta, EHP External heart power, E_a_ arterial load, E_max_ the slope of the end-systolic pressure-volume relationship, IHP Internal heart power. LV Left ventricle, PV-loops Pressure-Volume loops.

Heart work is characterized by internal (IHP) and external heart power (EHP). Heart power analysis was based on MRI data for the anatomy and 4D velocity field of the left ventricle such as catheter and peripherally measured cuff pressures. The acquired data (anatomy, pressure and flow curves) were combined to assess a cardiac energy profile (Fig 2), covering external and internal heart power (EHP and IHP), the power efficiency of the left ventricle defined as a relationship between EHP/IHP*100 [%], and data derived from pressure-volume loops.

Calculation of the EHP was based on MRI flow data and peripheral blood pressures using the following equation: *EHP* = *MAP* * *Q* [*W*], where MAP is the mean arterial pressure calculated from systolic and diastolic pressures assessed in the left arm and Q is the mean flow rate (cardiac output) assessed in the ascending aorta.

While catheter pressures significantly increase after the treatment procedure itself (due to pain or vascular reaction) the peripheral blood pressure values more accurately represent treatment outcome in the patient days after treatment. Therefore peripheral blood pressures, acquired during the MRI examination, were used to calculate EHP.

The calculation of the IHP was done according to the formula:

$$IHP=\frac{{V_{wall}*\sigma }_{wall}}{t_{CS}}$$

where V_wall_ is myocardial wall volume, *σ*_*wall*_ wall stress, and t_cs_ systolic contraction time of the left ventricle [17]. The wall stress calculation was condensed to the assessment of three parameters:

$$\sigma_{wall}=p_{sys}*\frac{R_{BP}}{2*S_{wall}}$$

where S_wall_ is averaged myocardial wall thickness, p_sys_ is the peak systolic pressure in the left ventricle and R_BP_ is the blood pool mean radius measured in a short axis sequence.

The contraction time t_CS_ can be assessed in two different ways: directly measured from the left ventricle pressure curve or calculated as:

$$t_{CS}=t_{IC}+t_{AC}$$

where t_AC_ is the time between diastolic and peak systolic pressures measured from the ascending aorta pressure curve, and t_IC_ is the isovolumetric contraction time calculated with follow assumptions:

- Left ventricle peak systolic pressure p_sys_ corresponds to the parabola vertex and is the sum of the ascending aorta peak systolic pressure plus peak systolic aortic valve pressure Δp_valve_,
- Peak systole pressure drop Δp_valve_ over aortic valve in pre- and post-treatment is the same since no significant differences were found between pre- and post-treatment peak systole flow rates and
- Left ventricular end-diastolic pressure is the same pre- and post-treatment.

Using two points of the parabola (peak systole as a vertex and diastolic pressure in the aorta with a period between these two points) the missing isovolumetric contraction time t_IC_−time between end-diastolic pressure and diastolic pressure as a start of the ejection–could be easily calculated.

Furthermore, data from pressure-volume loops were used to assess left ventricular contractility E_max_ and the arterial load E_a_. The slope of the end-systolic pressure-volume relationship (E_max_) was defined as a load-independent index of myocardial contractility. E_max_ (in mmHg/mL) was determined from catheter pressure data and MRI-derived volume loops via single beat estimation [18, 19]. The efficiency of coupling between the LV and the aorta was defined as the ratio of E_a_ to E_max_ [19].

### Statistical methods

Data are presented as means ± standard deviation, unless stated otherwise. All data were tested for normality using the Kolmogorov-Smirnov test. Normally distributed data were analyzed using the paired Student t-test for two-group comparisons (pre- vs. post-treatment). Associations between heart power and pressure gradients were assessed by linear regression. A repeated measures general linear model (GLM) was used for the assessment of IHP that takes into consideration a within-subject factor (the time point before and after an interventional procedure) as well as between-subjects effects as a group factor (increased IHP at the initial assessment). Prior to GLM, tests for normal distribution, sphericity and the Levene test were performed. Categorical data was tested using Fisher’s test. In this study, Bonferroni correction was used to adjust for multiple comparisons and differences were considered significant if p<0.0167. STATA Version 14.1 (StataCorp, Texas, USA) was used for the statistical analysis.

## Results

Of all 25 patients undergoing interventional treatment, 20 underwent full MRI reassessment with advanced segmentation and individual heart power analysis. In total 40 measurement points were used (n = 20 cases measured initially and at follow-up) with complete data sets available in all cases (S1 and S2 Tables). Of these patients 11 received stenting and 9 balloon angioplasty.

Table 2 summarizes geometric and hemodynamic parameters of the LV measured at the end-systolic phase from cine-MRI data as well as hemodynamic parameters before and after intervention. We found no significant differences in the parameters related to the LV geometry and hemodynamics. Systolic and diastolic blood pressure did not significantly change after intervention. However, changes in systolic blood pressure could be observed in individual patients and in MAP across all patients. At the time of the follow-up no significant reduction in the number of patients with systolic hypertension was observed.

**Table 2:** End-systolic geometric parameters (LVESD–left ventricular end-systolic diameter), and hemodynamic measures (LV–left ventricle).

	Before treatment	After treatment	p-value
**Left ventricular geometric parameters**			
Myocardial wall thickness [mm]	13.9±2.9	14.2±2.6	**0.530**
LVESD [mm]	28.3±6.9	28.2±5.7	**0.929**
Myocardial volume [mm^3^]	120.5±47.7	120.9±42.4	**0.956**
**Hemodynamic parameters**			
Heart rate [bpm]	75±15	76±22	**0.688**
Cardiac output [L/min]	5.5±1.35	5.4±1.54	**0.817**
Cardiac index [L/min*m^2^]	3.56±0.83	3.44±0.64	**0.518**
Systolic blood pressure [mmHg]	138.4±17.9	131.2±18.5	**0.052**
Diastolic blood pressure [mmHg]	69.3±16.3	67.2±12.4	**0.463**
Mean arterial pressure [mmHg]	89.5±16.2	82.7±13.5	**0.044**
Systolic hypertension	13 (65%)	12 (60%)	**0.744**
Cuff pressure gradient [mmHg]	20.2±21.4	7.2±17.3	**0.043**
Catheter pressure gradient [mmHg]	21.8(±9.4)	6.2 (± 6.1)	**<0.001**
LV Contractility (Emax) [mmHg/ml]	2.75±1.27	2.54±1.12	**0.445**
Elastance (Ea) [mmHg/ml]	1.17±0.5	1.38±0.63	**0.100**
Coupling Efficiency Ea/Emax [%]	45.3±16.15	70.5±60.7	**0.073**

**Fig 3:**
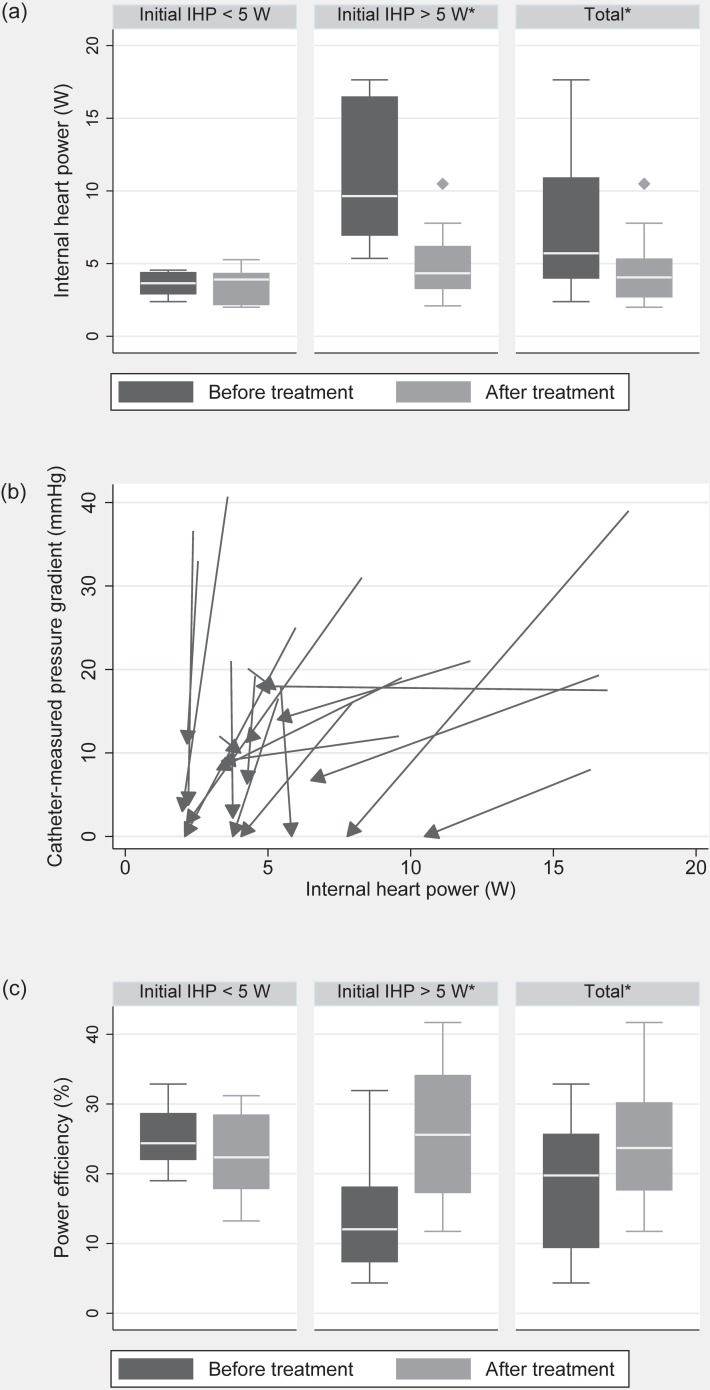
Main results. (a) Internal heart power (IHP) before and after treatment divided into groups according to the initial IHP above and below 5 W (b) The change in IHP with treatment plotted against the change of catheter-measured pressure gradients in each patient (the start-point of each vector represents the state before treatment, whereas the end-point represents the state after treatment) (c) Power efficiency before and after treatment grouped according to the initial IHP. * statistically significant differences.

Fig 3A shows changes of the internal heart power after treatment in the whole study population as well as in subgroups that were defined based on the initial IHP. Internal heart power was significantly reduced after intervention, from 8.03±5.2 to 4.37±2.13W (p < 0.001, Fig 3A). The variability before intervention was twice as high. While in patients initially presenting with IHP above 5W (n = 12) intervention resulted in a significant reduction (before 10.99±4.74W, after treatment 4.94±2.45W, p < 0.001), those presenting with IHP <5W (n = 8) did not undergo any significant changes after intervention (3.6±0.83W before vs. 3.51±1.23W after treatment, p = 0.746). A model-based repeated measures analysis resulted in significant independent effects of treatment (p<0.001) as well as group allocations according to IHP (p = 0.002).

Comparing the catheter gradient to the computed IHP no significant correlation was found. No correlation between IHP and the change (reduction) of the gradient was found. Fig 3B visualizes patient-specific changes of the IHP after the treatment as a 2D vector plot representing IHP (x-axis) and pressure gradients across the coarctation (y-axis). The start-point of each vector is a state before treatment, whereas the end-point represents the state after treatment. No association of IHP to gender or age was seen; however, the study population was not stratified accordingly.

The EHP was 1.1±0.3W before and 1±0.3W after intervention (p = 0.044). In those with an initial IHP > 5W a significant increase of the left ventricular efficiency (EHP/IHP) from 14 to 26% was observed (p = 0.005). We found a significant increase (p = 0.001) of the t_VC_ from 0.169±0.064s to 0.267±0.081s, also attributed to the subsequent decrease in the internal heart power after the treatment. No significant changes in the myocardial wall stresses were found (before 9.81±3kPa, after treatment 9.14±2.42kPa, p = 0.196).

Analysis of the E_a_, E_max_ and E_a_/E_max_ ratio before and after the treatment showed no significant differences in E_max_ (2.75±1.27 vs. 2.54±1.12 mmHg/ml, p = 0.445), the E_a_ (1.17±0.50 vs. 1.38±0.63mmHg/ml, p = 0.100) or E_a_/E_max_ ratio (0.45±0.16 vs. 0.70±0.61, p = 0.073).

## Discussion

It was successfully demonstrated that interventional treatment of CoA resulted in a significant decrease in IHP and EHP. Patients with higher IHP (>5W) show a significant reduction after treatment while those with low IHP (<5W) are likely to retain their initial IHP. Significantly reduced EHP can be seen as positive outcome in patients as the heart pumps blood against lower afterload pressure (MAP) after the relief of stenosis. The increase in power efficiency (EHP/IHP) in those patients with an initial IHP > 5W is mostly attributed to the reduction in IHP. Although the pressure gradient was significantly reduced in the overall study cohort, many patients remained hypertensive shortly after the procedure. The initial pressure gradient was not associated with (a) the IHP or (b) the reduction of IHP after interventional treatment. Current guidelines include both, a recommendation to reduce a pressure gradient and a more general stipulation of an optimal hemodynamic situation [1, 2]. Considering this, the analysis of a cardiac energy profile in CoA shows that these two aims are not necessarily linked and could even interfere with each other. A lack of knowledge in CoA exists regarding the exact influences on IHP and its independency of a relevant pressure gradient.

Different parameters have been described to assess heart power. They traditionally include the pressure-volume relationship (PV loops) [11] as well as derived parameters including stroke work and power efficiency, ventricular elastance and the slope of the end-systolic pressure volume diagram (E_max_−myocardial contractility). Further parameters such as power loss or energy dissipation and kinetic energy obtained from a non-invasive 4D-VENC MRI were also proposed to analyze ventricle dysfunction. Some of the proposed parameters are based on analytical considerations or computational fluid dynamics simulations. Lee et al. recently reviewed very detailed different energy-based approaches in congenital heart diseases affecting the right ventricle [12]. Older patients with chronic heart failure seem to present symptoms related to an impairment of cardiac and peripheral muscle metabolism and energetic phosphate production [20]. While no clear reference values exist in the assessment of heart power EHP can be easily derived from cardiac output and mean arterial pressure resulting in approximately 1 W in adults (presuming cardiac output of 5l/min and a blood pressure of 130/80 mmHg). Taking into account a normal myocardial power efficiency of 20–30% [17] normal IHP can be approximated as between 4 and 5 W. However, further research is required to determine the exact (disease-specific) reference ranges, and allow accurate classifications.

Cardiac MRI and catheterization are used as gold standards to assess ventricular function in clinical settings. To overcome limitations of these methods energy-based parameters (e.g. stroke work, efficiency and energy loss) have been proposed. Lee et al. 2015 discussed a set of energy based approaches based on 4D MRI analytical method or CFD to analyze the ventricle [12]. Additionally to internal and external heart power kinetic energy was explored. The method was earlier found to be able to show the impact of the treatment on the ventricular function [21]. We found, however, no significant differences between pre- and post-treatment kinetic energy curves.

A major challenge of the study was the assessment of IHP after treatment due to the absence of the post-treatment LV pressure data and to a stated increase in the pressures during the treatment procedure. To solve this problem, the systolic pressure in the aorta was calculated from the cuff pressure measured in the arm, whereas the diastolic pressures in the aorta and in the arm were considered to be equal [22]. The method of the contraction time calculation was validated using pre-treatment data of all cases–t_CS_ was directly measured from LV pressure curves (t_CS_meas_) and calculated (t_CS_calc_) according to the method described in the materials and methods. The linear regression analysis found significant correlation between calculated and measured LV contraction times t_CS_calc_ = 1.0529*t_CS_meas_ with a linear constant very close to 1.0 and a coefficient of determination r^2^ = 0.825.

The study is limited by a relatively low number of cases with both, measured catheter pressures and a complete follow-up assessment (of 52 eligible patients 25 patients undergoing interventional treatment and 20 patients with complete follow-up assessment) with data allowing calculation of the heart power. A statistical study power calculation using a statistical test of differences between two dependent means (matched pairs) resulted in an actual power of 0.9 in a total sample size of 15 and an effect size of 0.8. Furthermore, the study is associated with some limitations due to methodological, physiological and algorithmic uncertainties, which could have affected the measured parameters. The follow-up period was short and does not allow any reliable conclusions about long-term effects of treatment. As the baroreflex in patients with aortic coarctation has been described to be altered [23] it remains unknown whether the same effects apply as in other interventionally treated hypertensive patients. Recently, blood pressure reduction in patients after renal denervation was shown to occur as a gradual decrease that extends to at least one-year follow-up [24]. However, the problem of persisting hypertension in CoA at longer-term intervals has been demonstrated before and choosing longer follow-up periods in the assessment of heart power carries the risk of bias from re-stenosis, especially after balloon angioplasty [10, 25].

The analysis of IHP and EHP is further affected by the temporal and spatial resolution of MRI imaging and the accuracy in pressure measurements. The acquisition of the contraction time based on catheter measured pressures in the left ventricle was in most cases influenced by the double peak artefact present at the peak systole. To overcome this, we additionally analyzed simultaneously acquired ECG signals and tested the resulting values for the contraction time by comparison with the period (time between diastole and peak systole) assessed in the ascending aorta. This period is always shorter than the contraction time of the ventricle due to the isovolumetric contraction phase.

Conductance catheters currently serve as a reference standard to assess pressure volume loops. Although volume flow was obtained by MRI, pressure measurements are currently typically based on invasive catheterization in order to compute pressure volume loops, carrying the risks of non-simultaneous assessment. In order to avoid post-interventional catheterization artefacts, where the MAP is often influenced by a the treatment procedure itself due to pain and vascular response [26], cuff pressures were used for the calculation of EHP. The integrity of the left subclavian artery was ensured by MR imaging. By integration of the contraction time alongside the image acquisition process, additional information such as IHP and EHP will soon be available to clinicians based exclusively on non-invasive methods. Additional longer-term follow-up studies will contribute to a better understanding of the disease progression and pathophysiology. Subsequent dynamic disease models in CoA are highly warranted, as morbidity and mortality often remain high even years after the reduction of the actual narrowing.

## Conclusion

Pressure gradients, as the most widespread clinical parameters in CoA, did not show any correlation to IHP. The same is true for the reduction of a gradient, which is considered a major goal in interventional treatment: In patients in whom gradients were successfully reduced no correlation to IHP was found, raising the question of whether the reduction of pressure gradients should be the only focus of CoA interventions if they (a) cannot safely reduce the risk of arterial hypertension and (b) do not necessarily lead to optimization of the energy expenditure of the heart in all patients. The systematic assessment of internal heart power can help in identifying those patients with the greatest potential for improvements.

These findings are promising for clinical decision making and the assessment of functional myocardial remodeling in patients with CoA, and can potentially be of use in other cardiac diseases. Parameters can already be additional input in patient and disease specific predictions. Although reference values of IHP, EHP and power efficiency can be derived from normal cardiac physiology further studies will be required for validation and to explore the impact of abnormalities on the tissue level and on long term patient outcome.

## Supporting Information

S1 Protocol Clinical trial protocol (German original). (PDF)

S2 Protocol Clinical trial protocol (English computer translation). (PDF)

S1 Table TREND checklist. (PDF)

S2 Table Anonymized minimal data set. (XLS)

S3 Table Anonymized full data set. (XLS)
