# Correction: Beyond Pressure Gradients: The Effects of Intervention on Heart Power in Aortic Coarctation

**DOI:** 10.1371/journal.pone.0174838

**Published:** 2017-03-23

**Authors:** The PLOS ONE Staff

Figs 2 and 3 are displayed incorrectly in the PDF. The publisher apologizes for these errors. The authors have provided the corrected Figs 2 and 3 here.

**Fig 2:**
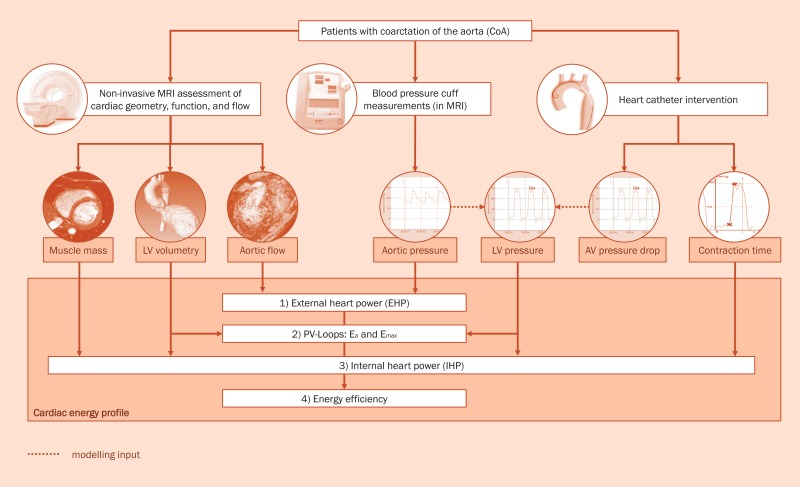
The assessment of a cardiac energy profile in patients with aortic coarctation. In all evaluated study participants this profile was acquired before and after the interventional treatment procedure. AV Aortic valve, CoA coarctation of the aorta, EHP External heart power, E_a_ arterial load, E_max_ the slope of the end-systolic pressure-volume relationship, IHP Internal heart power. LV Left ventricle, PV-loops Pressure-Volume loops.

**Fig 3:**
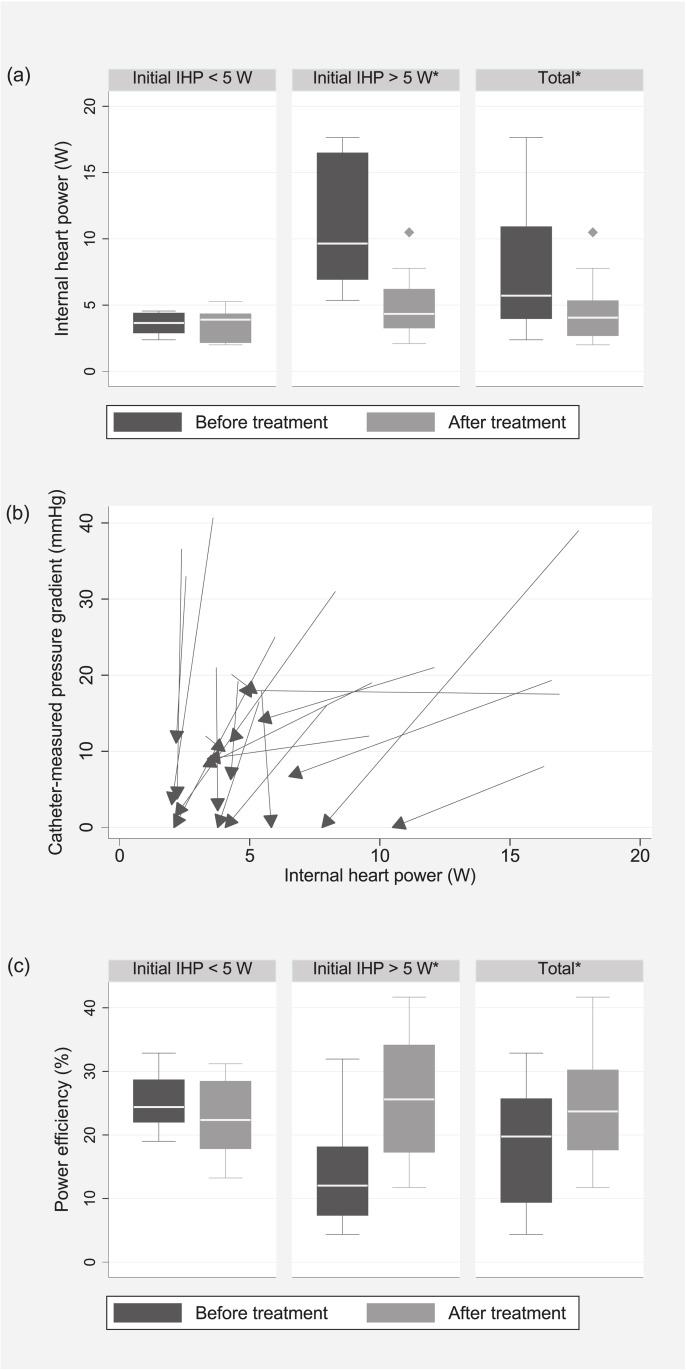
Main results. (a) Internal heart power (IHP) before and after treatment divided into groups according to the initial IHP above and below 5 W (b) The change in IHP with treatment plotted against the change of catheter-measured pressure gradients in each patient (the start-point of each vector represents the state before treatment, whereas the end-point represents the state after treatment) (c) Power efficiency before and after treatment grouped according to the initial IHP. * statistically significant differences.
