# Supplementary material for: Beyond Pressure Gradients: The Effects of Intervention on Heart Power in Aortic Coarctation
Source: PLoS One. 2017 Jan 12;12(1):e0168487. doi: 10.1371/journal.pone.0168487 (PMC5231370; doi:10.1371/journal.pone.0168487)
Supplement: S1 Protocol — (PDF) [file pone.0168487.s001.pdf]

Antrag auf Beratung durch die Ethikkommission zur Durchführung eines medizinisch-wissenschaftlichen Vorhabens, welches weder die klinische Prüfung eines Arzneimittels oder Medizinproduktes beinhaltet

1. Titel der Studie	Computer unterstützte Analyse und Vorhersage des hämodynamischen Ergebnisses von Behandlungsstrategien bei Herzfehlern
2. Ethikkommissions -Antragsnummer	*(wird von der EK vergeben)*
3. Entscheidungen anderer Ethikkommissionen in derselben Sache	Keine, jedoch bestehen große Überschneidungen zu den von der Ethikkommission der Charité genehmigten Anträgen EA2/018/09 sowie EA2/124/07 und v.a. **EA2/110/10** (vom 10.11.2010)
4. Gegenstand der Studie und ihre Ziele; Angabe der Hypothesen, getrennt in Haupt- und Sekundärhypothesen sowie der klinischen Parameter (primäre und sekundäre Endpunkte), anhand derer die Hypothesen geprüft werden	Gegenstand der Studie ist die Analyse und Vorhersage des hämodynamischen Ergebnisses von Interventionen bei Patienten mit Herzfehler, insbesondere Stenosen des Aortenisthmus,der Pulmonalarterien und der Aortenklappe. Hierzu sollen Bildbasierte (MRT) Modellierungen der Funktion erfolgen<br>Hypothese: Die Bildbasierte Modellierung soll Angaben über Flussmuster und Druckfelder in den betroffenen Gefäßabschnitten liefern. So soll zum einen die diagnostische Herzkatheteruntersuchung ersetzt werden, zum anderen die hämodynamischen Effekte vor dem Eingriff simuliert und damit langfristig optimiert werden
5. Erläuterung der Bedeutung der Studie	**Bei Stenosen der Pulmonalarterien und des Aortenisthmus sind häufig im Verlauf des Wachstums herzkatheterbasierte Eingriffe, z.B. Ballon-Dilatationen oder Stent-Implantationen, notwendig. Bei Aortenklappenvitien ist es wichtig, den richtigen Zeitpunkt für einen Klappenersatz zu finden, da die Klappenhaltbarkeit begrenzt ist (Vermeidung von zu frühen Eingriffen) und gleichzeitig die Entstehung einer Herzinsuffizienz vermieden werden soll (Vermeidung zu später Eingriffe). Bei sämtlichen Eingriffen ist es zudem wichtig, möglichst physiologische Verhältnisse zu schaffen, da ansonsten das Risiko für Re-Interventionen steigt.**<br>**Mit Hilfe der geplanten Studie sollen nun Methoden überprüft werden, mit denen sich die hämodynamischen Effekte eines Eingriffs vorab simulieren lassen (Bildbasierte Modellierung).**
6. Welche der folgenden Bestimmungen finden Anwendung<br>a) Medizinproduktegesetz gemäß § 23 b MPG - Ausnahme der klin. Prüfung<br>b) Strahlenschutzverordnung § 23<br>c) Röntgenverordnung § 28 a<br>d) Gendiagnostikgesetz<br>e) Datenschutzgesetze:<br>- Konkrete Angabe des durch die verantwortliche Stelle zu erfüllenden Datenschutzgesetzes (für die Charité = Berliner Datenschutzgesetz - BlnDSG).<br>- Ggf. entsprechend des Teilnehmerkreises zusätzlich zu beachtende Landesdaten-	e) das Datenschutzgesetz

schutzgesetze oder BDSG.	
7. Ggf.: Bezeichnung und Charakterisierung der Prüfprodukte	Entfällt
8. wesentliche Ergebnisse der vorklinischen Tests oder Gründe für die Nichtdurchführung derselben	Vorklinische Tests existieren nicht, da die Anatomien der stenotischen Gefäße oder Herzklappen als Modelle nicht existieren.
9. Wesentlicher Inhalt und Ergebnisse der vorangegangenen Studien/Anwendungen der in der Studie zu prüfenden Produkte	Entfällt
10. Beschreibung der vorgesehenen Maßnahmen/Untersuchungsmethoden und eventuelle Abweichungen von den in der med. Praxis üblichen Maßnahmen/Untersuchungen (was ist „Routine", was wird davon abweichend in der Studie gemacht?)	Vorhandene MRT-Bilddaten sowie Druckwerte von Herzkatheteruntersuchungen, die im Rahmen der Routine-Diagnostik gewonnen wurden, sollen für die bildbasierte Modellierung verwendet werden.<br>Bei volljährigen Patienten ist mit deren Zustimmung eine MRT-Untersuchung nach einem Eingriff (Herzkatheter oder OP) ohne Verwendung von Kontrastmittel angedacht. Sollte sich nach erfolgtem Eingriff (Herzkatheter oder OP) eine klinische Indikation für eine MRT-Verlaufskontrolle ergeben, sollen diese Daten ebenfalls der Analyse zugeführt werden.
11. Bewertung und Abwägung der vorhersehbaren Risiken und Nachteile der Studienteilnahme gegenüber dem erwarteten Nutzen für die Studienteilnehmer und zukünftig erkrankte Personen (Nutzen-Risiko-Abwägung)	Nachteile sind nicht zu erwarten.
a. Voraussehbarer therapeutischer Nutzen für die Studienteilnehmer (individueller Nutzen für den einzelnen Patienten)	Durch die Modellierung ist auch für die teilnehmenden Patienten für zukünftige evtl. notwendig werdende Re-Eingriffe ein Nutzen zu erwarten, da idealerweise invasive diagnostische Herzkatheterisierungen überflüssig werden. Zudem können optimierte Ergebnisse nach Herzkatheterintervention/OP erwartet werden.
b. Voraussehbarer medizinischer Nutzen für zukünftig erkrankte Personen (Gruppennutzen)	Bei zukünftig erkrankten Personen können idealerweise diagnostische Herzkatheteruntersuchungen durch die MRT ersetzt werden. Zudem können optimierte Ergebnisse nach Herzkatheterintervention/OP erwartet werden.
c. **Risiken** und Belastungen für die Studienteilnehmer (alle im Einzelnen auflisten)	Die MRT-Untersuchung ohne Kontrastmittelanwendung hat bei Einhaltung der Sicherheitsrichtlinien keine erwiesenen Risiken.
12. Maßnahmen zur Risikobeherrschung	Entfällt
13. Abbruchkriterien	Falls Patienten bzw. Sorgeberechtigte die Teilnahme an der Studie widerrufen

14. Anzahl, Alter und Geschlecht der betroffenen Personen	20 Patienten mit Pulmonalstenosen,40 Patienten mit Aortenisthmusstenose, 60 Patienten mit Aortenklappen Vitium, unterschiedlichen Alters beiderlei Geschlechts
15. Biometrische Planung mit Angabe der statistischen Methodik, einschließlich der Begründung der Fallzahl.<br>Angabe des/der Statistikers/Statistikerin	Entfällt, da es sich um eine Pilotstudie handelt.
16.<br>a. Darlegung und ggf. Erläuterung der **Ein- und Ausschlusskriterien**	Eingeschlossen werden sollen Patienten mit Pulmonalstenosen (Pulmonalisstamm, rechte oder linke Pulmonalarterie) bzw. Aortenisthmusstenosen bzw. Aortenklappenvitien, bei denen MRT-Bilddaten vorliegen bzw. eine MRT-Untersuchung indiziert ist.
b. **Teilnehmerinformation** (wer diese mündlich und schriftlich erteilt und Angabe, wie viel Zeit zwischen Aufklärung und Einwilligung verbleibt (schriftliche Information als Anlage)	Mündlich durch Studienleiter oder Studienarzt sowie schriftliche Teilnehmerinformation (siehe Anlage)
c. **Einwilligungserklärung** (schriftliche Form als Anlage)	siehe Anlage
d. Ggf. **Information und Einwilligung des gesetzlichen Vertreters** (ggf. auch Beschreibung des Verfahrens zur Einrichtung einer gerichtlichen Betreuung)	siehe 16.c
17. Maßnahmen zur Gewinnung von Studienteilnehmern (Aushang ?, Zeitungsannoncen? Etc.)	Rekrutierung aus dem aktuellen bzw. zukünftigen Patientengut
18. Ggf.: **Grund für die Einbeziehung und Darlegung des therapeutischen Nutzens für Personen, die minderjährig und/oder nicht einwilligungsfähig sind.**	Bei Herzfehlern sind Gefäßstenosen (z.B. Aortenisthmus) aber auch Aortenklappenfehler (bicuspide Aortenklappe) auch im Kindesalter häufig, und im Verlauf des Wachstums sind notwendige Interventionen nicht selten.
19. Beziehung zwischen Studienteilnehmer und Studienarzt/-ärztin (Ist der Studienarzt zugleich der behandelnde Arzt?)	Der Studienarzt ist auch behandelnder Arzt.
20. Erklärung zur Einbeziehung möglicherweise vom Sponsor abhängiger Personen	Entfällt
21. Maßnahmen, die eine Feststellung zulassen, ob ein Studienteilnehmer an mehreren Studien zugleich oder vor Ablauf einer in der vorangegangenen Studie festgelegten Frist teilnimmt.<br>Ist die Teilnahme an mehreren Studien möglich?	Gegen eine gleichzeitige Teilnahme an einer anderen Studie ist nichts einzuwenden.
22. Ggf.: Honorierung bzw. Kostenerstattung der Studienteilnehmer (Höhe, wofür soll gezahlt werden?)	Entfällt
23. Ggf.: Plan für die Weiterbehandlung und medizinische Betreuung der betroffenen Personen nach dem Ende der Studie	Patienten werden kontinuierlich medizinisch und (kinder-) kardiologisch betreut.

24. Ggf.: Versicherung der Studienteilnehmer (Versicherungsbestätigung und Versicherungsbedingungen, Versicherer, Versicherungsumfang, Versicherungsdauer)	Keine zusätzliche Versicherung über die sowieso bestehende hinaus
25. Dokumentationsverfahren:<br>- Ggf. Verweis auf CRF-Bögen.<br>- Detaillierte Angabe der zu erfassenden personenbezogenen Daten.<br>- Angabe von Datenkategorien (Studiendaten).<br>- Erhebungsart (Papier, digital, digital beim Empfänger).<br>- Probenumgang<br>- Aufbewahrung / Archivierung (inkl. Fristen)<br>- Zugang zu den Daten und Proben	Siehe eCRF-Bogen für Patienten (Anlage)
26. Ggf.: Beschreibung, wie der Gesundheitszustand gesunder betroffener Personen dokumentiert werden soll	Entfällt
27. Ggf.: Methoden, unerwünschte Ereignisse festzustellen, zu dokumentieren und mitzuteilen (wann, von wem und wie ??)	Entfällt
28. Vorgehen zum Schutz der Geheimhaltung der gespeicherten Daten, Dokumente und ggf. Proben, Darlegung der Pseudonymisierung oder Anonymisierung der Daten und Proben von Studienteilnehmern **(Initialen und Geburtsdatum als Codierungsschema sind nicht zulässig!)**<br>- Beschreibung der Trennung von Krankenakten, Studiendokumentation und Zuordnung der personenbezogenen Daten<br><br>- Nennung der Zugriffsrechte einschließlich des Zugangs zu Teilnehmeridentifikationslisten während und nach der Studiendurchführung<br>- Detaillierte Angabe der Verfahren für die Übertragung, Verschlüsselung, Sperrung und Löschung (einschließlich Angabe der ggf. verwendeten Netzstruktur und verwendete Server).	Jedem Studienteilnehmer wird bei Einschluss in die Studie einmalig eine Nummer (ID) zugelost. Für die Auswertung der studienassoziierten MRT-Ergebnisse und Herzkatheter-Befunde werden jeweils nur solche Daten verwendet, bei denen die personenbezogenen Merkmale (Name, Vorname, Geschlecht, Geburtsdatum, Anschrift) durch diese ID ersetzt worden sind.<br>Die Studiendokumentation betreffende Unterlagen werden getrennt von den Krankenakten in einem abgeschlossenen Schrank aufbewahrt.<br>Zugriff auf die Studiendokumentation einschließlich der Teilnehmeridentifikationslisten haben der Studienleiter sowie von ihm beauftrage Mitarbeiter, welche Studienärzte und/oder -schwestern sein können.<br>Die studienbezogenen Daten (Bilddaten, Untersuchungsergebnisse) werden auf Forschungsservern des DHZB gespeichert (Intranet). Die Teilnehmeridentifikationslisten werden auf einem separaten Rechner gespeichert, zu dem nur der Studienleiter und von ihm beauftragte Mitarbeiter Zugang haben.
29. Erklärung zur Einhaltung des Datenschutzes<br>- Zusicherung, dass alle über den Studienteilnehmer erhobenen und gespeicherten Daten vertraulich (Datengeheimnis und ärztliche Schweigepflicht) behandelt werden.<br>- Zusicherung, dass die identifizierenden Daten nur dem Studienleiter oder von ihm beauftragten Mitarbeitern zugänglich sind.<br>- Angabe der Maßnahmen zur Sicherstellung der Vertraulichkeit<br>- Maßnahmen zur datenschutzgerechten Übermittlung von Daten, die für Dritte	Personenbezogene Daten (Name, Vorname, Geschlecht, Geburtsdatum, Anschrift) einschließlich Krankheits- und Gesundheitsdaten, die im Rahmen dieser Studie erhoben werden, sowie die Ergebnisse der studienassoziierten zusätzlichen Untersuchungen werden im Falle einer Studienteilnahme folgendermaßen verarbeitet:<br>Der aufklärende Studienarzt erhebt zunächst die personenbezogenen Daten und vermerkt diese auf der Einwilligungserklärung, anschließend wird ein Kennwort (Pseudonym) gebildet. Alle studienassoziiert erhobenen und gewonnenen Gesundheits- und Krankheitsdaten sowie die Ergebnisse der Studie werden zusammen mit dem zuvor gebildeten Pseudonym gespeichert. Der

keinen Personenbezug herstellen lassen.<br>- Angabe der Auskunfts- Widerspruchs- und Löschmöglichkeiten,<br>- Maßnahmen zur Sicherstellung der Rechte der Teilnehmer.	Schlüssel, um diese Daten den personenbezogenen Daten zuzuordnen, wird auf einem separaten Computer von den übrigen Daten getrennt gespeichert. Dieser ist nur dem Studienleiter bzw. von ihm beauftragen Mitarbeitern zugänglich. Nach Archivierung entsprechend der gesetzlichen Aufbewahrungsfrist werden die Studiendaten vernichtet bzw. gelöscht.<br>Die Publikation der Studienergebnisse erfolgt anonym.
30. Namen und Anschriften der Einrichtungen, die als Studienzentrum oder Studienlabor in die Studie eingebunden sind, sowie der Studienleiter und die Studienärzte<br>- Detaillierte Angabe beteiligter externer Dienstleister mit Angabe der Datenzugriffsmöglichkeit.	Studienzentrum:<br>Deutsches Herzzentrum Berlin (DHZB) und Charité Berlin, Abteilung Kinderkardiologie und Angeborene Herzfehler, Nicht-invasive Bildgebung<br>Augustenburger Platz 1<br>13353 Berlin<br><br>Studienleiter: Prof. Dr. Titus Kühne<br>Studienärzte: Dr . Marcus Kelm<br>Lucio Biocca
31. Angaben zur Eignung der Prüfstelle, insbesondere zur Angemessenheit der dort vorhandenen Mittel und Einrichtungen sowie des zur Durchführung der klinischen Prüfung zur Verfügung stehenden Personals und zu Erfahrungen in der Durchführung ähnlicher Studien	Am DHZB und der Charité werden zahlreiche Patienten mit angeborenem und erworbenem Herzfehler mittels MRT und im Herzkatheter untersucht. Zur MRT-Bildgebung stehen modernste Geräte zur Verfügung.
32. Vereinbarung über den Zugang des Prüfers/Hauptprüfers/Leiter der klinischen Prüfung, zu den Daten und den Grundsätzen über die Publikation.<br>- Publikationen in einer Form, die keinen Rückschluss auf die Person zulassen.<br>- ggf. Zugang zu identifizierenden Daten für die<br>gesetzlich berechtigten Prüfer (Dritte) zur zweckgebundenen Einsichtnahme in die dafür erforderlichen Akten.	Der Studienleiter und die von ihm beauftragten Mitarbeiter haben uneingeschränkten Zugriff auf die im Rahmen der Studie erhobenen Daten.<br>Entscheidungen bezüglich der Publikation der Daten obliegen dem Studienleiter. Dabei erfolgt die Publikation stets anonym.
33. Angaben zur Finanzierung der Studie: Finanzierungsquelle (Name und Sitz)	Das Projekt CARDIOPROOF wird von der Europäischen Kommission, Brüssel gefördert.

Name und Unterschrift des/der Antragstellers:

Ich versichere hiermit, dass die in diesem Antrag gegebenen Informationen richtig sind. Ich bin der Auffassung, dass es möglich ist, die o.g. Studie in Übereinstimmung mit dem Protokoll, den nationalen Rechtsvorschriften durchzuführen.

Mir ist bekannt, dass ich gemäß §19 Berliner Datenschutzgesetz (BlnDSG) verpflichtet bin, für automatisierte Verarbeitungen personenbezogener und personenbeziehbarer Daten eine Datei- und Verfahrensbeschreibung zu erstellen und diese gemäß §19a dem behördlichen Datenschutzbeauftragten der Charité zur Verfügung stellen muss. Ich bin darüber informiert, dass wenn es sich um ein Verfahren handelt, mit dem Daten verarbeitet werden, die einem Berufsgeheimnis (z.B. ärztliche Schweigepflicht) unterliegen, ich gemäß §5 BlnDSG vor dem Einsatz dieses Verfahrens eine Vorabkontrolle durch den behördlichen Datenschutzbeauftragten der Charité veranlassen muss und ich das Verfahren erst bei positivem Prüfergebnis anwenden darf.

Name: Prof. Dr. Kühne

Vorname: Titus

Adresse: Augustenburger Platz 1, 13353 Berlin

Position: Leiter der Abteilung Nicht-invasive Bildgebung bei Angeborenen Herzfehlern, DHZB-Charité

Datum: 11. November 2013

Unterschrift: [illegible], Berlin 12.11.13

Die Zustimmung des Klinikdirektors Prof. Dr. Felix Berger, Direktor der Abteilungen für Angeborene Herzfehler und Kinderkardiologie der Charité sowie des DHZB, liegt vor.

Unterschrift: [illegible]
