# Supplementary material for: Beyond Pressure Gradients: The Effects of Intervention on Heart Power in Aortic Coarctation
Source: PLoS One. 2017 Jan 12;12(1):e0168487. doi: 10.1371/journal.pone.0168487 (PMC5231370; doi:10.1371/journal.pone.0168487)
Supplement: S2 Protocol — (PDF) [file pone.0168487.s002.pdf]

Ethical proposal for the implementation of a medical-scientific project (which is neither a pharmacological trial nor includes market approval of a medical product).

1. Title of the study	Computer-aided analysis and prediction of the hemodynamic result of treatment strategies for heart disease
2. Ethics Commission -request number	*(is assigned by the EK)*
3. Decisions of other ethics committees in the same case	None, but there is a large overlap of the ethics commission of the Charité approved applications EA2/018/09 and EA2/124/07 and especially **EA2/110/10** (10.11.2010)
4. The purpose of the study and its objectives; indication of the hypotheses, separated into primary and secondary hypotheses as well as the clinical parameters (primary and secondary endpoints), on the basis of which the hypotheses checked	The purpose of the study is the analysis and prediction of the hemodynamic result of interventions in patients with heart disease, particularly stenosis of the aortic isthmus, the pulmonary arteries and the aortic valve. To this end, image-based (MRI) modeling of the function<br>Hypothesis: The image-based modeling can predict hemodynamic parameters. Thus, for a diagnostic heart catheter examination could be replaced, and the hemodynamic effects before the intervention can be simulated and optimized, aiming towards optimized outcome.
5. Explanation of the importance of the study	**In stenosis of the arteries and the aortic isthmus are often in the course of growth of heart catheter-based interventions, e.g. balloon dilatations or stent placement, necessary. In aortic valve disease it is important to choose the right timing for a valve replacement, as the valve durability is limited (prevention of early intervention) and thus avoide the emergence to heart failure (avoid later complications and reinterventions). For all interventions, it is also important to achieve physiological conditions, because otherwise the risk for re-interventionen increases.**<br>**With the help of the planned study we will investigate methods with which the hemodynamic effects of an intervention can be simulated (e.g. image-based modeling).**
6. Which of the following terms apply<br>a) Medical products law pursuant to § 23 b MPG - the exception of Klin. Test<br>b) Radiation Protection Regulation § 23<br>c) X-ray Regulation § 28 a<br>d) Gene diagnostics law<br>e) Data protection laws:<br>- concrete indication of the by the Responsible Body to fulfill Data Protection Act (for the Charité Berlin = Data Protection Act - BlnDSG).<br>- if necessary, according to the circle of participants in addition to consider national data protection laws or the BDSG).	(e) the Data Protection laws

7. If necessary: description and characterisation of the test Products	n.a.
8. The essential results of pre-clinical tests or the reasons for not carrying out the same	Pre-clinical tests do not exist since the anatomy of the stenotic vessels or heart valves are not available.
9. Substantial content and results of the previous studies/applications of research in the study of products to be tested	n.a.
10. Description of the planned measures/methods of investigation and any deviations from the practice in the med. Usual measures/Studies (what is "routine", what is different in the study done?)	MR image data as well as the pressure values of heart catheter investigations are carried out in the framework of routine diagnostics will be used for the image-based modeling.<br>In patients consenting an MRI exam after an intervention (heart catheter or surgery) will be scheduled without the use of contrast agent. If after intervention (heart catheter or surgery) a clinical indication for an MRI control exists, any of these data is also be supplied to the analysis.
11. Evaluation and consideration of the foreseeable risks and disadvantages of study participation against the expected benefits for the study participants and will in future be ill persons (benefit-risk assessment)	Disadvantages are not to be expected.
A. Predictable therapeutic benefit for the study participants (individual benefits for the individual patient)	By modeling, participating patients could benefit from gain of knowledge for the future if re-interventions are neccessary and ideally invasive diagnostic heart catheterization. In addition, heart catheter intervention/surgical procedures could be optimized in the future.
As Foreseeable medical benefit for the future in the diseased persons (group use)	In future diseased patients diagnostic heart catheter examinations can ideally be replaced by MRI. In addition, the optimized results after heart catheter intervention/OP are expected.
C). **Risks** and burdens to the study participants (all in each list)	The MRI study without contrast agent application is in compliance with the safety guidelines, and is without any proven risks.
12. Measures for risk control	n.a.
13. Abort Criteria	If patients or custody participation in the study revoked
14. Number, age and sex of the persons concerned	20 patients with pulmonary stenosis,40 patients with coarctation of the aorta, 60 patients with aortic valves disease, undergoing treatment. of different ages and different genders
15. Biometric planning with specification of the statistical methodology, including the justification for the case number.<br>Indication of the statistician/Statistics Erin	n.a., because it is a pilot study.

16.<br>A. Presentation and any explanation of the **inclusion and exclusion criteria**	Inclusion criteria were any of the following diseases: pulmonary stenosis, aortic coarctation, aortic aortic valve disease in which MRI image data are available with the intervention
Participant **information** (presented orally and in writing and specify how much time remains between information and Consent (written information as an attachment)	Orally by the leader of the study or study physicians as well as written participant information and informed consent (see appendix)
C). **A Declaration of Consent** (written form as attachment)	See Appendix
D. If necessary **information and consent of the legal representative** (if necessary, also the description of the procedure for the establishment of a judicial support)	See 16.c
17. Measures for the recovery of study participants (Notifications, announcements etc).	Recruitment from the current or future patient collective
18. If necessary: **the reason for the inclusion and explanation of the therapeutic benefits for persons under age of 18 and/or the need for consent by a legal representative**.	In congenital heart disease stenoses of the vessles (e.g., coarctation), and aortic valve disease (bicuspid aortic valve disease) are frequent congenital heart diseases, and often require interventions in children and in the course of growth are necessary.
19. Relationship between study participants and study doctor/doctor (is the study doctor at the same time the doctor?)	The study physician is also the treating physician.
20. Declaration on the inclusion of possible from the Sponsor dependent persons	n.a.
21. Measures which allow a determination as to whether a study participants can be participating in several studies at the same time or before the expiry of a specified in the previous study period takes part<br>is the participation in several studies possible?	A simultaneous participation in another study is possible.
22. If necessary: payment or reimbursement of the study participants (height, what should be paid?)	n.a.
23. If necessary: Plan for the further treatment and medical care to the persons concerned after the end of the study	Patients are continuously and medically an in cardiologic care.
24. If necessary: Insurance of the study participants (insurance confirmation and insurance conditions, insurers, scope of insurance, insurance duration)	No additional insurance besides the existing anyway
25. Documentation procedures:<br>- if necessary, reference to CRF arc.<br>- Detailed description of the to be acquired Personal Data.<br>- Specification of the categories of data (studies and data).	eCRF-sheet for patients

- collection type (paper, digital, digital when Receiver).<br>- Sample Handling<br>- storage/archiving (incl. deadlines)<br>- access to data and samples	
26. If necessary: description, such as the state of health of healthy person affected should be documented	n.a.
27. If necessary: methods, adverse events noted, to document and communicate (when, by whom and how??)	n.a.
28. Procedure to protect the confidentiality of stored data, documents and any samples, presentations of pseudonym coding or anonymization of the data and samples of study participants **(initials and date of birth as the encoding scheme are not allowed!)**<br>- a description of the separation of medical records, study documentation and assignment of the personal data<br><br>- the access rights including access to participant identification lists during and after the study by management<br>- Detailed description of the procedure for the transmission, encryption, blocking and deletion (including an indication of any network structure and used server).	On inclusion each participant will be assigned a unique and randomly generated number (ID). For the evaluation of the study associated MRI results and heart catheter findings are only such data used in which the personal characteristics (Name, First Name, Sex, Date of birth, address) by this ID have been replaced.<br>The study documentation records are separate from the medical records in a locked cabinet.<br>Access to the study documentation including the Participant Identification lists are available to the study leader and instructed employees, which can be study physicians and/or nurses.<br>The study-related data (image data, test results) will be stored on research servers within the DHZB (Intranet). The participant identification lists are stored on a separate computer to which only the study leader and IT staff have access.
29. Declaration on compliance with the Data Protection<br>- assurance that all collected data are stored confidential (respecting data secrecy and medical confidentiality legislation).<br><br>- assurance that the identifying Data only the study director or his delegate employees are accessible.<br>- indication of the type of measures to ensure the confidentiality<br>- measures for data protection of transfer of data to third parties no personal reference.<br>- indication of the type of information the extinguishing capabilities<br>- measures to ensure the rights of the participants.	Personal data (First Name, Last Name, Gender, date of birth, address) including disease specific and health care data, in the course of this study are to be collected and the results of the study are associated additional investigations in the event of a study participation are processed as follows:<br>The study physician to whom consent will be fiven first assesses the personal data and records this in the declaration of consent, then a pseudonym will be assigned. All associated study collected and acquired health and disease data as well as the results of the study will be used together with the previously formed pseudonym stored. The key to this data is on a separate computer stored separately on another computer. This is only available to the study leader or instructed employees. After archiving according to the legal retention period, the study data are destroyed or deleted. The publication of the findings of the study will be anonymous at all times.
30. The names and addresses of the bodies which a study center or study laboratory in the study are involved, as well as the director of studies and the study doctors<br>- Detailed information about external stakeholders<br>Service provider with specification of the data access	Study Center:<br>German Heart Center Berlin (DHZB) and Charité Berlin, Department of Paediatric Cardiology and Congenital Heart Disease, non-invasive imaging<br>Augustenburger Platz 1<br>13353 Berlin<br><br>Director of studies: Prof. Dr. Titus Kuhne

Possibility.	Study physicians: Dr . Marcus Kelm<br>Lucio Biocca
31. Information on the suitability of the inspection body, in particular regarding the appropriateness of the existing resources and facilities as well as the conduct of the clinical trial available personnel and experience in the implementation of similar studies	At the DHZB and the Charité many patients with congenital and acquired heart disease are being examined using MRI, as well as in the heart catheter lab. For MRI imaging state-of-the-art equipment isavailable.
32. Agreement on access of auditor/principal auditor/head of the clinical examination, to the data and the principles concerning the publication .<br>- publications in a form that no conclusion on the individual.<br>- if necessary the access to specific data for the<br>Legally justified auditor (third parties) to Dedicated access to the Necessary files.	The study leader and commissioned staff have access to in the framework of the study collected data. Decisions regarding the publication of the data are the responsibility of the director of studies. The publication is always anonymous.
33. Details of the financing of the study : Funding Source (Name and Seat)	The project CARDIOPROOF is funded by the European Commission, Brussels.

Name and signature of the applicant:

I hereby declare that the information given in this application is correct. I am of the opinion that it is possible that the above study in accordance with the protocol that the national legislation.

I know that I in accordance with §19 Berlin Data Protection Act (BlnDSG) am committed, for automated processing of personal and personobtainable Daten a file and description of methods to create and according to §19a the data protection officer of the Charité has to provide. I am informed that if it is a procedure that allows the data to be processed to a professional secrecy (e.g. medical confidentiality), i according to §5 BlnDSG before using this procedure a prior checking by the Data Protection Officer of the Charité and i must arrange for the procedure only in the case of a positive test result may apply.

Name: Prof. Dr. Kuhne

First name: Titus

Address: August citizens Platz 1, 13353 Berlin

Position: Head of the department of non-invasive imaging for congenital heart defects, DHZB-Charité

Date: 11. November 2013

Signature:

The approval of the clinic director Prof. Dr. Felix Berger, Director of the department for congenital heart defects and pediatric cardiology the Charité and the DHZB.

Signature:
